# Amygdala Cannabinoid 1 Receptor, Pain Response, and Emotional Numbing in Trauma-Exposed Individuals

**DOI:** 10.1001/jamanetworkopen.2024.32387

**Published:** 2024-09-09

**Authors:** Nachshon Korem, Ansel T. Hillmer, Deepak Cyril D’Souza, Anahita Bassir Nia, Ifat Levy, Robert H. Pietrzak, Ilan Harpaz-Rotem

**Affiliations:** 1 Department of Psychiatry, Yale University School of Medicine, New Haven, Connecticut; 2 Department of Comparative Medicine, Yale University School of Medicine, New Haven, Connecticut; 3 US Department of Veterans Affairs National Center for Posttraumatic Stress Disorder, VA Connecticut Healthcare System, West Haven, Connecticut; 4 Department of Radiology and Biomedical Imaging, Yale School of Medicine, New Haven, Connecticut; 5 Department of Biomedical Engineering, Yale School of Engineering and Applied Sciences, Yale University, New Haven, Connecticut; 6 Wu Tsai Institute, Yale University New Haven, New Haven, Connecticut; 7 Department of Psychology, Yale University, New Haven, Connecticut; 8 Department of Neuroscience, Yale University, New Haven, Connecticut

## Abstract

This case-control study assesses associations of amygdala cannabinoid 1 receptor availability with amygdala response to shock-induced pain and severity of emotional numbing symptoms of veterans with posttraumatic stress disorder.

## Introduction

Exposure to traumatic events profoundly alters the processing of physiological and emotional pain.^1,2^ Recently, we showed reduced amygdala responses to mild pain (electric shocks) in veterans with posttraumatic stress disorder (PTSD).^1^ This diminished response was associated with greater severity of emotional numbing (EN) symptoms (eg, restricted affect). Given the crucial role of the endocannabinoid (eCB) system in pain modulation,^3^ and stress-related disorders such as PTSD,^4^ we hypothesized that the eCB system may play a role in this response. To test this hypothesis, we examined the association of amygdala CB1 receptor (CB1R) availability, a critical node in the eCB system with (1) amygdala response to shock-induced pain and (2) severity of EN symptoms of PTSD.

## Methods

In this case-control study, trauma-exposed, non–cannabis-smoking adults underwent clinical assessments, including the Clinician-Administered PTSD Scale for the *Diagnostic and Statistical Manual of Mental Disorders, Fifth Edition* (CAPS-5). Then, CB1R availability was measured using a positron emission tomography (PET) scan and the radioligand [^11^C]OMAR.^5^ Participants completed a fear-conditioning task during a functional magnetic resonance imaging scan to evaluate the amygdala response to mild pain (mean/median [SD/IQR] time between scans, 13.00 [13.26] days).^1^

Ethical approval and written consent from all participants were obtained. The study was approved by the Yale University institutional review board and adhered to STROBE reporting guidelines.

CB1R availability was quantified in the amygdala using [^11^C]OMAR total volume of distribution estimated from PET data.^5^ Amygdala response to shocks was extracted from the contrast between the conditioned stimulus paired with the unconditioned stimulus and the conditioned stimulus alone, utilizing a predefined amygdala mask.^1^ Severity of EN symptoms was calculated by summing items 12 to 14 of the CAPS-5. Associations were tested using bayesian robust linear (activation) and 0-inflated Poisson (symptoms) regression analysis adjusted for sex, Z-transformed age, and Z-transformed body mass index (see eMethods in Supplement 1 for detailed methods and sensitivity analysis). Results were considered robust if 0 fell outside the highest density posterior (HPD). Statistical analyses were performed from April to May 2024 in Python version 3.9.13 (Python Software Foundation) using PyMC version 4.1.7 and ArviZ (version 0.12.1) packages.^2^

## Results

The study sample consisted of 30 trauma-exposed adults (mean [SD] age, 44.4 [14.5] years; 6 female [20%]), including 9 with a current PTSD diagnosis. A robust negative association of amygdala CB1R availability with response to shock was observed (mean posterior distribution, −0.76; 89% HPD, −1.22 to −0.31) (Figure, A). In addition, a robust positive association of amygdala CB1R availability and severity of EN symptoms (mean posterior distribution, 0.70; 89% HPD, 0.09 to 1.31) (Figure, B). No other cluster was associated with CB1R (Table).

**Figure.**
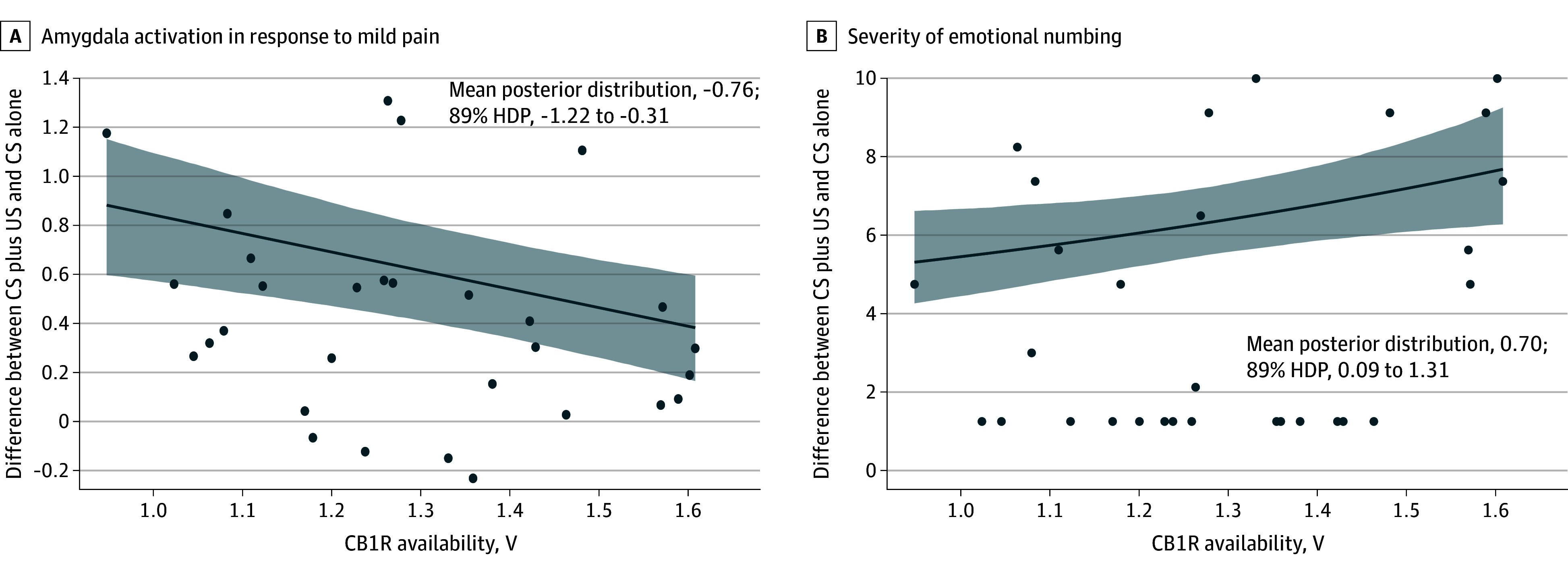
Cannabinoid Receptor 1 (CB1R) Availability in the Amygdala and Amygdala Activation to Shocks and Emotional Numbing Severity A, Robust regression analysis examined the association of CB1R availability in the amygdala with amygdala activation in response to mild pain. We utilized Logan graphical analysis with metabolite-corrected arterial input functions to derive the total volume of distribution as the primary outcome measure. B, 0-Inflated Poisson robust regression assessed the association of CB1R availability in the amygdala with the severity of emotional numbing. All models exhibited convergence (R-hat < 1.01; effective sampling rate > 1000). Markov Chain Monte Carlo inference employed the no-U-turn sampler, following PyMC default settings (1000 draws; 1000 tuning steps; 80% acceptance rate without thinning). CS indicates conditioned stimulus; HDP, highest density posterior; US, unconditioned stimulus.

**Table.** Cannabinoid Receptor 1 Availability in the Amygdala and Posttraumatic Stress Disorder Symptom Clusters

Factor	Slope, mean (89% HDP)
Internally generated intrusion symptoms	−0.47 (−1.24 to 0.30)
Externally generated intrusion symptoms	0.08 (−0.70 to 0.84)
Avoidance	0.33 (−0.41 to 1.13)
Negative affect	0.14 (−0.50 to 0.81)
Emotional numbing	0.71 (0.10 to 1.33)
Externalizing behaviors	−0.12 (−0.95 to 0.67)
Anxious arousal	0.43 (−0.26 to 1.18)
Dysphoric arousal	−0.10 (−0.84 to 0.62)

Abbreviation: HDP, highest density posterior.

## Discussion

Results of this case-control study show a robust negative association of amygdala CB1R availability with amygdala response to shock-induced pain among trauma-exposed adults. This finding suggests that increased amygdala CB1R availability, which is indicative of lower eCB tone,^6^ may contribute in part to diminished pain responsiveness in trauma survivors. Additionally, we identified a robust positive association of amygdala CB1R availability with the severity of EN symptoms. These findings support our hypothesis that increased CB1R availability modulates amygdala response to pain and increased severity of EN symptoms.^1,2^

The brain stress response involves eCB release,^3^ which primarily inhibits the release of other neurotransmitters. Given the high density of CB1R in the amygdala, trauma-exposed individuals are primed for lower responsiveness to mild noxious stimuli, thus inducing an emotionally numb state among trauma survivors. Reduced responsiveness to mild stimuli may be associated with hyper-responsiveness to more aversive stimuli.^1,2^

These findings are intriguing yet limited; they need to be replicated in a larger sample along with other measures of the eCB system, such as anandamide and 2-arachidonoylglycerol levels and the levels of fatty acid amide hydrolase and monoacylglycerol lipase to capture the state of eCB signaling fully. Lastly, these findings also raise the possibility of harnessing the therapeutic potential of the eCB system to study the association of physiological pain with emotional pain.

## Notes

Supplement 1.
eMethods.

eReferences.

Supplement 2.
Data Sharing Statement
