# Supplementary material for: Amygdala Cannabinoid 1 Receptor, Pain Response, and Emotional Numbing in Trauma-Exposed Individuals
Source: JAMA Netw Open. 2024 Sep 9;7(9):e2432387. doi: 10.1001/jamanetworkopen.2024.32387 (PMC11385051; doi:10.1001/jamanetworkopen.2024.32387)
Supplement: Supplement 1. — eMethods. eReferences. [file jamanetwopen-e2432387-s001.pdf]

Supplemental Online Content

Korem N, Hillmer AT, D'Souza DC, et al. Amygdala cannabinoid 1 receptor, pain response, and emotional numbing in trauma-exposed individuals. *JAMA Netw. Open*. 2024;7(9):e2432387. doi:10.1001/jamanetworkopen.2024.32387

eMethods
eReferences

This supplemental material has been provided by the authors to give readers additional information about their work.

© 2024 Korem N et al. *JAMA Network Open.*

eMethods

Screening

All participants underwent clinical screening using the Structured Clinical Interview for DSM-V Axis I Disorders (SCID-V)[1] and the Clinician-Administered PTSD Scale for DSM-V (CAPS-V).[2] The Institutional Review Boards of Yale University and the VA Connecticut Healthcare System approved the study. All participants gave informed consent and received monetary compensation for their participation. Two participants filled out the PTSD checklist for DSM-5 (PCL-5)[3] instead of the CAPS-5.

Electric Shock Calibration

The shock was delivered using two electrodes positioned on the inner wrist of the participant's dominant hand, connected to a Constant Voltage Stimulator—Unipolar Pulse (Model STM200; Biopac Systems, Inc., Goleta, CA). Shock levels were individually adjusted for each participant. Beginning at a minimal shock level (20 volts), the experimenter incrementally increased the intensity until participants reported it as "painful but tolerable," which then became the set level for the duration of the experiment.

Fear Reversal Task

Participants engaged in a fear reversal task.[4] In the task, participants were instructed to observe two colored squares (blue and yellow) displayed on a screen and evaluate their association with the likelihood of them co-terminating with an electric shock (Delay/Cue fear-conditioning). To control for order effects, the sequence of stimuli presentation was counterbalanced across participants. Initially, in the experiment's first phase, one color (CS+) was partially paired with the shock, with six presentations of CS+US and 12 of

© 2024 Korem N et al. *JAMA Network Open.*

CS+ alone (33% reinforcement rate). Meanwhile, the second stimulus (CS-) was not associated with the shock and was presented 12 times. Subsequently, without forewarning the participants, the contingency was reversed. In the reversal phase, the CS+ was no longer paired with the shock (16 presentations), while the CS- was now associated with the shock (7 CS-US and 16 CS- alone; 30% reinforcement rate).

Magnetic Resonance Imaging (MRI) Acquisition

MRI data were obtained using a 3 T Siemens Prisma scanner at the Yale Magnetic Resonance Research Center (MRRC), equipped with a 32-channel receiver array head coil. High-resolution structural images were acquired via Magnetization-Prepared Rapid Gradient-Echo (MPRAGE) imaging (TR = 2.5 s, TE = 2.83 ms, FOV = 256 × 256 mm^2, matrix = 256 x 256 mm^2, slice thickness = 1.0 mm without gap, 160 slices, voxel size 1.0 × 1.0 × 1.0 mm^3). Functional MRI scans were conducted during the fear reversal task using a multi-band (4) Echo-planar Imaging (EPI) sequence (TR = 1000 ms, TE = 30 ms, flip angle = 60°, voxel size = 2 × 2 × 2 mm^3, 60 2 mm-thick slices, in-plane resolution = 2 × 2 mm^2, FOV = 220 mm).

fMRI Neural Data Preprocessing

Results included in this manuscript come from preprocessing performed using fMRIPrep 21.0.1,[5,6] which is based on Nipype 1.6.1.[7]

Anatomical Data Preprocessing

A total of 1 T1-weighted (T1w) images were found within the input BIDS dataset. The T1-weighted (T1w) image was corrected for intensity non-uniformity (INU) with N4BiasFieldCorrection,[8] distributed with ANTs 2.3.3,[9] and used as T1w-reference throughout the workflow. The T1w-reference was then skull-stripped with a Nipype implementation of the antsBrainExtraction.sh workflow (from ANTs), using OASIS30ANTs as the target template. Brain tissue segmentation of cerebrospinal fluid

© 2024 Korem N et al. *JAMA Network Open.*

(CSF), white matter (WM), and gray matter (GM) was performed on the brain-extracted T1w using fast (FSL 6.0.5.1:57b01774).[10] Brain surfaces were reconstructed using recon-all (FreeSurfer 6.0.1),[11] and the brain mask estimated previously was refined with a custom variation of the method to reconcile ANTs-derived and FreeSurfer-derived segmentations of the cortical gray-matter of Mindboggle.[12] Volume-based spatial normalization to two standard spaces (MNI152NLin2009cAsym, MNI152NLin6Asym) was performed through nonlinear registration with antsRegistration (ANTs 2.3.3), using brain-extracted versions of both T1w reference and the T1w template. The following templates were selected for spatial normalization: ICBM 152 Nonlinear Asymmetrical template version 2009c (TemplateFlow ID: MNI152NLin2009cAsym),[13] FSL's MNI ICBM 152 non-linear 6th Generation Asymmetric Average Brain Stereotaxic Registration Model (TemplateFlow ID: MNI152NLin6Asym).[14]

Functional Data Preprocessing

For each subject's BOLD run, the following preprocessing steps were implemented. First, a reference volume and its skull-stripped version were generated using a custom methodology of fMRIPrep. Head-motion parameters with respect to the BOLD reference (transformation matrices and six corresponding rotation and translation parameters) are estimated before any spatiotemporal filtering using mcflirt (FSL 6.0.5.1:57b01774).[15] BOLD runs were slice-time corrected to 0.458s (0.5 of slice acquisition range 0s-0.915s) using 3dTshift from AFNI.[16] The BOLD time series (including slice-timing correction when applied) were resampled onto their original, native space by applying the transforms to correct for head motion. These resampled BOLD time series will be called preprocessed BOLD in the original space or just preprocessed BOLD. The BOLD reference was then co-registered to the T1w reference using bbregister (FreeSurfer) which implements boundary-based registration.[17] Co-registration was configured with six degrees of freedom. Several confounding time series were calculated based on the preprocessed BOLD: framewise displacement (FD), DVARS, and three region-wise global signals. FD was computed using two formulations following Power (absolute sum of relative motions).[15,18] FD and DVARS are calculated for each functional run, both using their implementations in Nipype (following

© 2024 Korem N et al. *JAMA Network Open.*

the definitions by Power et al.).[18] The three global signals are extracted within the CSF, the WM, and the whole-brain masks. A set of physiological regressors was also extracted to allow for component-based noise correction (CompCor).[19] Principal components are estimated after high-pass filtering the preprocessed BOLD time series (using a discrete cosine filter with 128s cut-off) for the two CompCor variants: temporal (tCompCor) and anatomical (aCompCor). tCompCor components are calculated from the brain mask's top 2% variable voxels. For aCompCor, three probabilistic masks (CSF, WM, and combined CSF+WM) are generated in anatomical space. The implementation differs from that of Behzadi et al.[19] in that instead of eroding the masks by 2 pixels on BOLD space, the aCompCor masks subtract a mask of pixels that likely contain a volume fraction of GM. This mask is obtained by dilating a GM mask extracted from the FreeSurfer's ASEG segmentation, and it ensures components are not extracted from voxels containing a minimal fraction of GM. Finally, these masks are resampled into BOLD space and binarized by thresholding at 0.99 (as in the original implementation). Components are also calculated separately within the WM and CSF masks. For each CompCor decomposition, the k components with the largest singular values are retained, such that the retained components' time series are sufficient to explain 50 percent of variance across the nuisance mask (CSF, WM, combined, or temporal). The remaining components are dropped from consideration. The head-motion estimates calculated in the correction step were also placed within the corresponding confounds file. The confound time series derived from head motion estimates and global signals was expanded by including temporal derivatives and quadratic terms for each.[20] Frames that exceeded a threshold of 0.5 mm FD or 1.5 standardized DVARS were annotated as motion outliers. The BOLD time series were resampled into standard space, generating a preprocessed BOLD run in MNI152NLin2009cAsym space. First, a reference volume and its skull-stripped version were generated using a custom methodology of fMRIPrep. The BOLD time series were resampled onto the following surfaces (FreeSurfer reconstruction nomenclature): fsnative, fsaverage5, fsaverage. Grayordinates files containing 91k samples were also generated using the highest-resolution fsaverage as intermediate standardized surface space.[21] All resamplings can be performed with a single interpolation step by composing

© 2024 Korem N et al. *JAMA Network Open.*

all the pertinent transformations (i.e., head-motion transform matrices, susceptibility distortion correction when available, and co-registrations to anatomical and output spaces). Gridded (volumetric) resamplings were performed using antsApplyTransforms (ANTs), configured with Lanczos interpolation to minimize the smoothing effects of other kernels.[22] Non-gridded (surface) resamplings were performed using mri_vol2surf (FreeSurfer).

Many internal operations of fMRIPrep use Nilearn 0.8.1,[23] mostly within the functional processing workflow. For more pipeline details, see the workflow section in fMRIPrep's documentation.

First-Level fMRI Analysis

First-level analysis was performed using SPM. The BOLD signal for each subject was corrected for six confounds (rotation and translation), Framewise Displacement (FD), the standard deviation of DVARS, and the first six anatomical CompCor components. Additionally, we calculated the number of frames with FD greater than 0.5mm and excluded subjects with 40% or more frames exceeding this threshold; however, no subjects were excluded based on this criterion. Subsequently, we computed the CS+US > CS+ contrast to evaluate the impact of the unconditioned stimulus (US) on the amygdala. Nipype version 1.8.5.3 was utilized to conduct the SPM analysis.[7] A complete script of the analysis is available at https://github.com/KoremNSN/CB1Pain.

Radiochemistry and PET Image Acquisition

[^{11}C]OMAR was synthesized using established protocols adapted for automated production on the GE TRACERlab FXC-Pro synthesis module (GE Healthcare, Milwaukee, WI, USA).[24] Mean (SD) molar activity at time of injection was 167 (105) MBq/nmol and injected activity was 481 (153) MBq.

Participants underwent dynamic PET scans using an HRRT scanner (Siemens Medical Systems, Knoxville, TN). Prior to each PET scan, a transmission scan was acquired with a ^{137}Cs point source for attenuation correction. PET emission data acquisition

began with the administration of [^{11}C]OMAR via slow bolus intravenous infusion over 1 minute and continued for 120 minutes. Participant motion was monitored using an optical system (Polaris Vicra, Northern Digital Incorporated, Waterloo, Ontario, Canada) positioned behind the PET scanner. This system recorded the three-dimensional position and orientation of an infrared reflective tool mounted rigidly to the subject's head using a Lycra cap and Coban self-adherent wrap.

Radioactivity concentration in arterial whole blood was measured during the initial 7 minutes following [^{11}C]OMAR administration using either continuous measurement with an integrated peristaltic pump and radioactivity detection system (PBS101, Veenstra Instruments, Joure, The Netherlands) or rapid manual sampling. Discrete samples were manually drawn for all scans at specific time points (3, 5, 7, 10, 15, 20, 30, 45, 60, 75, 90, 105, and 120 minutes post-injection). Gamma counter measurements (Wizard 1480, PerkinElmer, Waltham, MA, USA) were used to determine each sample's whole blood and plasma radioactivity. This was then converted to concentration based on the sample weight and density. Additionally, samples collected at specific time points (5, 15, 30, 60, 90, and 120 minutes) were analyzed using column-switching high-performance liquid chromatography (HPLC)[25] to determine the fraction of unmetabolized radiotracer, as previously described.[26] The unmetabolized parent fraction was calculated as the ratio of radioactivity in fractions containing the parent compound to the total radioactivity collected, fitted with an inverted gamma function, and normalized by the time-varying extraction efficiency of radioactivity for the corresponding filtered plasma sample. Finally, the metabolite-corrected arterial plasma input function was obtained by multiplying the total plasma radioactivity concentration curve with the parent fraction curve on a point-by-point basis.

PET Image Processing

Dynamic scan data underwent reconstruction with corrections for subject motion, attenuation, normalization, scatter, randoms, and dead time using an ordered subset-expectation maximization (OSEM)[27] algorithm (2 iterations, 30 subsets) histogrammed into 33 frames. Software motion correction was performed on the dynamic images using

a mutual-information algorithm (FSL-FLIRT version 3.2, Analysis Group, FMRIB, Oxford, UK),[28] employing frame-by-frame registration to an early summed image (0-10 min post-injection), which was also registered to the subject's MR anatomical image (6-parameter affine registration). The subject-specific MR image was subsequently registered to the Anatomical Automatic Labeling (AAL)[30] atlas using a non-linear registration routine for region of interest (ROI) delineation.[29] The final outcome measure was regional [^{11}C]OMAR volume of distribution (V_T), referred to as CB1R availability because it is proportional to the number of CB1 receptors available for [^{11}C]OMAR binding.[31] [^{11}C]OMAR V_T was estimated using the multilinear analysis-1 method (MA1)[32] with a reference time (t*) of 30 minutes.

Statistical Analysis

In this study, we conducted a robust Bayesian regression analysis to investigate the relationship between amygdala and insula CB1R availability ([^{11}C]OMAR V_T) and participants' shock responses. The dependent variable was the average activation in the amygdala or insula, while CB1R availability in the respective regions served as the independent variable. Covariates included sex, z-transformed age, and z-transformed BMI. The analysis utilized partially informed priors

$$intercept \sim Normal\ (1,1)$$

$$V_t\ slope \sim Normal(0,1)$$

$$Sex \sim Normal(0,1)$$

$$Age\ Z \sim Normal(0,1)$$

$$BMI\ Z \sim Normal(0,1)$$

For the independent variable, a Student’s t distribution was employed to accommodate outliers

$$response\ to\ pain \sim Student's\ t\ (\mu, \nu, \varepsilon)$$

$$\nu \sim Inverse\ Gamma\ (3,1)$$

$$\varepsilon \sim Exponential\ (1)$$

We employed a Zero-inflated Poisson (ZIP) Robust Bayesian regression analysis to explore the association between amygdala CB1R availability (V_T) and PTSD symptom

© 2024 Korem N et al. *JAMA Network Open.*

clusters. Here, participants' symptom cluster score served as the dependent variable, while [^{11}C]OMAR V_T availability in the amygdala was the independent variable, with sex, z-transformed age, and z-transformed BMI as covariates. Results were exponentiated to ensure they remained positive. Similar partially informed priors were used:

$$intercept \sim Normal\ (1{,}1)$$

$$V_T\ slope \sim Normal(0{,}1)$$

$$Sex \sim Normal(0{,}1)$$

$$Age\ Z \sim Normal(0{,}1)$$

$$BMI\ Z \sim Normal(0{,}1)$$

The independent variable, representing the symptom cluster, was modeled using a Zero-inflated Poisson distribution to account for the influx of people reporting zero symptoms.

$$Cluster\ score \sim ZIP\ (\mu, \psi)$$

$$\psi \sim Beta\ (1, 1)$$

A robust association was considered if the 89% Highest Posterior Density (HPD) of the slope did not include 0. All models converge with rHat<1.01 and effective sampling rate > 1000. All analyses were conducted in Python 3.9.13, utilizing the 'PyMC' (version 4.1.7)[33] and 'ArviZ' (version 0.12.1)[34] packages. We employed the No-U-Turn Sampler (NUTS) for MCMC inference, adhering to PyMC's default settings: 1000 draws, 1000 tuning steps, and an 80% acceptance rate, without thinning.

© 2024 Korem N et al. *JAMA Network Open.*

eReferences

1. First MB, Williams JB, Karg RS, Spitzer RL. *User's Guide for the SCID-5-CV Structured Clinical Interview for DSM-5® Disorders: Clinical Version.* American Psychiatric Publishing, Inc.; 2016.
2. Weathers FW, Bovin MJ, Lee DJ, et al. The Clinician-Administered PTSD Scale for DSM–5 (CAPS-5): Development and initial psychometric evaluation in military veterans. *Psychol Assess*. 2018;30(3):383.
3. Blevins CA, Weathers FW, Davis MT, Witte TK, Domino JL. The posttraumatic stress disorder checklist for DSM-5 (PCL-5): Development and initial psychometric evaluation. *J Trauma Stress*. 2015;28(6):489-498.
4. Schiller D, Levy I, Niv Y, LeDoux JE, Phelps EA. From Fear to Safety and Back: Reversal of Fear in the Human Brain. *J Neurosci*. 2008;28(45):11517-11525. doi:10.1523/JNEUROSCI.2265-08.2008
5. Esteban O, Markiewicz CJ, Blair RW, et al. FMRIPrep: a robust preprocessing pipeline for functional MRI. *Nat Methods*. 2019;16(1):111-116. doi:10.1038/s41592-018-0235-4
6. Esteban O, Markiewicz CJ, Blair RW, et al. fMRIPrep: a robust preprocessing pipeline for functional MRI. *Nat Methods*. 2019;16(1):111-116. doi:10.1038/s41592-018-0235-4
7. Gorgolewski K, Burns CD, Madison C, et al. Nipype: a flexible, lightweight and extensible neuroimaging data processing framework in python. *Front Neuroinformatics*. 2011;5:13.
8. Tustison NJ, Avants BB, Cook PA, et al. N4ITK: improved N3 bias correction. *IEEE Trans Med Imaging*. 2010;29(6):1310-1320.
9. Avants BB, Epstein CL, Grossman M, Gee JC. Symmetric diffeomorphic image registration with cross-correlation: evaluating automated labeling of elderly and neurodegenerative brain. *Med Image Anal*. 2008;12(1):26-41.
10. Zhang Y, Brady M, Smith S. Segmentation of brain MR images through a hidden Markov random field model and the expectation-maximization algorithm. *IEEE Trans Med Imaging*. 2001;20(1):45-57.
11. Dale AM, Fischl B, Sereno MI. Cortical surface-based analysis: I. Segmentation and surface reconstruction. *Neuroimage*. 1999;9(2):179-194.
12. Klein A, Ghosh SS, Bao FS, et al. Mindboggling morphometry of human brains. *PLoS Comput Biol*. 2017;13(2):e1005350.
13. Fonov VS, Evans AC, McKinstry RC, Almli CR, Collins DL. Unbiased nonlinear average age-appropriate brain templates from birth to adulthood. *NeuroImage*. 2009;(47):S102.
14. Evans AC, Janke AL, Collins DL, Baillet S. Brain templates and atlases. *NeuroImage*. 2012;62(2):911-922. doi:10.1016/j.neuroimage.2012.01.024
15. Jenkinson M, Bannister P, Brady M, Smith S. Improved optimization for the robust and accurate linear registration and motion correction of brain images. *Neuroimage*. 2002;17(2):825-841.
16. Cox RW, Hyde JS. Software tools for analysis and visualization of fMRI data. *NMR Biomed Int J Devoted Dev Appl Magn Reson Vivo*. 1997;10(4-5):171-178.
17. Greve DN, Fischl B. Accurate and robust brain image alignment using boundary-based registration. *Neuroimage*. 2009;48(1):63-72.
18. Power JD, Mitra A, Laumann TO, Snyder AZ, Schlaggar BL, Petersen SE. Methods to detect, characterize, and remove motion artifact in resting state fMRI. *Neuroimage*. 2014;84:320-341.
19. Behzadi Y, Restom K, Liau J, Liu TT. A component based noise correction method (CompCor) for BOLD and perfusion based fMRI. *Neuroimage*. 2007;37(1):90-101.
20. Satterthwaite TD, Elliott MA, Gerraty RT, et al. An improved framework for confound regression and filtering for control of motion artifact in the preprocessing of resting-state functional connectivity data. *NeuroImage*. 2013;64:240-256.

© 2024 Korem N et al. *JAMA Network Open.*

doi:10.1016/j.neuroimage.2012.08.052
21. Glasser MF, Sotiropoulos SN, Wilson JA, et al. The minimal preprocessing pipelines for the Human Connectome Project. *Neuroimage*. 2013;80:105-124.
22. Lanczos C. Evaluation of noisy data. *J Soc Ind Appl Math Ser B Numer Anal*. 1964;1(1):76-85.
23. Abraham A, Pedregosa F, Eickenberg M, et al. Machine learning for neuroimaging with scikit-learn. *Front Neuroinformatics*. 2014;8:14.
24. Horti AG, Fan H, Kuwabara H, et al. 11C-JHU75528: a radiotracer for PET imaging of CB1 cannabinoid receptors. *J Nucl Med*. 2006;47(10):1689-1696.
25. Hilton J, Yokoi F, Dannals RF, Ravert HT, Szabo Z, Wong DF. Column-switching HPLC for the analysis of plasma in PET imaging studies. *Nucl Med Biol*. 2000;27(6):627-630. doi:10.1016/S0969-8051(00)00125-6
26. Normandin MD, Zheng MQ, Lin KS, et al. Imaging the Cannabinoid CB1 Receptor in Humans with [11C] OMAR: Assessment of Kinetic Analysis Methods, Test–Retest Reproducibility, and Gender Differences. *J Cereb Blood Flow Metab*. 2015;35(8):1313-1322. doi:10.1038/jcbfm.2015.46
27. Carson RE, Barker WC, Liow JS, Johnson CA. Design of a motion-compensation OSEM list-mode algorithm for resolution-recovery reconstruction for the HRRT. In: *2003 IEEE Nuclear Science Symposium. Conference Record (IEEE Cat. No. 03CH37515)*. Vol 5. IEEE; 2003:3281-3285.
28. Jenkinson M, Smith S. A global optimisation method for robust affine registration of brain images. *Med Image Anal*. 2001;5(2):143-156.
29. Papademetris X, Jackowski MP, Rajeevan N, et al. BioImage Suite: An integrated medical image analysis suite: An update. *Insight J*. 2006;2006:209.
30. Tzourio-Mazoyer N, Landeau B, Papathanassiou D, et al. Automated anatomical labeling of activations in SPM using a macroscopic anatomical parcellation of the MNI MRI single-subject brain. *NeuroImage*. 2002;15(1):273-289. doi:10.1006/nimg.2001.0978
31. Innis RB, Cunningham VJ, Delforge J, et al. Consensus nomenclature for in vivo imaging of reversibly binding radioligands. *J Cereb Blood Flow Metab*. 2007;27(9):1533-1539.
32. Ichise M, Toyama H, Innis RB, Carson RE. Strategies to improve neuroreceptor parameter estimation by linear regression analysis. *J Cereb Blood Flow Metab*. 2002;22(10):1271-1281.
33. Abril-Pla O, Andreani V, Carroll C, et al. PyMC: a modern, and comprehensive probabilistic programming framework in Python. *PeerJ Comput Sci*. 2023;9:e1516.
34. Kumar R, Carroll C, Hartikainen A, Martín OA. ArviZ a unified library for exploratory analysis of Bayesian models in Python. Published online 2019.

© 2024 Korem N et al. *JAMA Network Open.*
