# Supplementary material for: Amygdala Cannabinoid 1 Receptor, Pain Response, and Emotional Numbing in Trauma-Exposed Individuals
Source: JAMA Netw Open. 2024 Sep 9;7(9):e2432387. doi: 10.1001/jamanetworkopen.2024.32387 (PMC11385051; doi:10.1001/jamanetworkopen.2024.32387)
Supplement: Supplement 2. — Data Sharing Statement [file jamanetwopen-e2432387-s002.pdf]

Data Sharing Statement

Korem. Amygdala Cannabinoid 1 Receptor, Pain Response, and Emotional Numbing in Trauma-Exposed Individuals. *JAMA Netw Open*. Published September 09, 2024. doi:10.1001/jamanetworkopen.2024.32387

Data
Data available: No

Additional Information
Explanation for why data not available: clinical data cannot be shared
